# Quality and Educational Value of YouTube Surgical Videos of Transurethral Resection of the Prostate: A Descriptive Cross-Sectional Study

**DOI:** 10.7759/cureus.68141

**Published:** 2024-08-29

**Authors:** Mohammed Elsiddig, Mohammed Hassan

**Affiliations:** 1 Urology, Military Hospital, Khartoum, SDN

**Keywords:** lap-vegas, laparoscopic surgery video educational guidelines, turp, transurethral resection of the prostate, benign prostate hyperplasia

## Abstract

Introduction

The aim of the study is to evaluate the quality and educational value of surgical videos on YouTube (Alphabet Inc., Mountain View, CA) demonstrating transurethral resection of the prostate (TURP).

Methods

A thorough YouTube search for "TURP" or "transurethral resection of the prostate" was performed. Each video's uploader, content, duration, date of upload, time since upload, views, comments, likes, and dislikes, and Video Power Index (VPI) scores were recorded and evaluated. Video analysis and rating followed the LAParoscopic Surgery Video Educational Guidelines (LAP-VEGaS) recommendations, which constitute nine items with values from 0 (absence) to 2 (complete presence). The guidelines' overall score can be 0 to 18. A higher score is indicative of a better level of educational value.

Results

There were a total of 43 videos included, 10 (23.3%) of which were academic publications. The average LAP-VEGaS score was 6.58, with 22 (51.2%), 18 (41.8%), and three (7%) videos classified as having low, medium, and high educational quality, respectively. None of the videos satisfied all the requirements outlined in the checklist. There was no statistically significant positive correlation observed between the educational score and the number of views.

Conclusion

A significant proportion of transurethral resection of the prostate (TURP) videos available on the YouTube platform exhibit limited educational value. Videos frequently lack comprehensive and in-depth descriptions of surgical operations. Those seeking information on TURP should carefully choose which videos to view. It is recommended that academic institutions establish comprehensive criteria aimed at enhancing the educational value of surgical videos on the YouTube platform.

## Introduction

YouTube (Alphabet Inc., Mountain View, CA) is one of the most widely used online video-sharing platforms due to its accessibility, user-friendliness, and wealth of content [1, 2]. It provides cost-effective, convenient, and familiar communication to learners and is available 24 hours a day, seven days a week. Its extensive collection of medical and surgical videos has contributed to its recent increase in use in medical education [2]. The rising prevalence of YouTube will likely present opportunities and challenges for current medical education paradigms [3]. According to studies, medical students in multiple surgical specialties have utilized this platform for educational purposes [4].

Some educators argue that YouTube can enhance learning opportunities outside the classroom and is particularly useful for promoting self-directed learning [5]. Several published studies characterize the educational value of YouTube for medical trainees in various specialties [6]. Clifton and Mann explain why YouTube benefits education and directs attention to essential information: "Visual delivery is a well-established technique for retaining information." According to previous research, using social media improves self-regulated learning and enables students to participate in the learning process at any time and in any location. In addition, it facilitates collaborative and communicative learning by permitting learners to interact, remark, and provide feedback on videos [7].

On the other hand, some authors have cautioned that the content of educational videos on social media could complicate medical education, which is responsible for teaching and training medical professionals. For instance, poor quality, lack of professionalism, patient confidentiality issues, and absence of references seen in some of the available video content have raised concerns [7, 8].

Transurethral resection of the prostate (TURP) is the standard surgical treatment for benign prostatic hyperplasia [9, 10]. TURP is also one of the cornerstones of every urology training curriculum. Various entities, including individual surgeons, academic societies, and commercial enterprises, have uploaded a multitude of TURP surgical videos on YouTube.

YouTube could serve as a potential educational resource for urology trainees learning about transurethral resection of the prostate (TURP) due to its video-based nature; however, there is limited data regarding the educational quality of TURP videos uploaded to YouTube. The educational value of these videos remains uncertain due to the paucity of peer review and quality assessment [11, 12]. To our knowledge, no published study has evaluated the quality and utility of TURP surgical videos.

This study aims to evaluate the educational utility of TURP surgical videos on YouTube. Furthermore, this study has the potential to facilitate the development of a comprehensive educational video checklist specifically tailored for TURP surgery. The study will also aid novices in identifying valuable TURP videos on YouTube. We hypothesize that the number of views of a video may not be proportional to its educational value.

## Materials and methods

This is a descriptive, cross-sectional assessment of YouTube videos as a potential educational tool in urology education. We conducted searches on YouTube (http://www.youtube.com) using the terms "TURP" or "transurethral resection of the prostate." We filtered the videos based on their view counts, selecting the 65 most-viewed videos for analysis. We excluded videos that featured non-surgical content like patient education, 3-D animation, or hospital advertisements. Also excluded were videos demonstrating TURP in addition to other procedures, such as litholapaxy (crushing and removal of urinary bladder stones).

We analyzed basic characteristics such as the duration, number of views, number of comments, and number of likes and dislikes for each selected video. We determined the popularity of the videos by evaluating their view-to-like ratio using the "Video Power Index" (VPI). After calculating the like ratio (like*100/[like+dislike]) and the view ratio (number of views/days), the VPI equals the like ratio*view ratio/100 [13, 14].

For each video, we evaluated the educational content, surgical technical aspects, and compliance with the LAParoscopic Surgery Video Educational Guidelines (LAP-VEGaS). Using the LAParoscopic Surgery Video Educational Guidelines (LAP-VEGaS), a validated surgical video assessment tool, the researcher evaluated the quality of TURP videos. Each item in this instrument receives a rating of either not present (0), moderately present (1), or fully present (2). The sum of the scores on each of the nine items, which range from 0 to 18, is the total result. The videos were classified as low, medium, or high quality based on their designated scores (low: 0-6, medium: 7-12, and high: 13-18) [15-17].

We used the LAP-VEGaS guidelines to analyze the content of each video and determine any correlations with the identified characteristics. By correlating LAP-VEGaS with these characteristics, we aimed to assess whether there existed any discernible relationship between video content and these specific attributes.

We entered the data into Microsoft Excel (Microsoft Corp., Redmond, WA) spreadsheets and analyzed it using SPSS Statistics (IBM Corp., Armonk, NY). Descriptive statistics were reported as means, medians, and standard deviations for continuous and ordinal variables, and as frequencies (n) and percentages (%) for categorical variables. The correlations between variables were evaluated using the Pearson and Spearman correlation coefficients. The Mann-Whitney U test was used to compare LAP-VEGaS scores for videos published by academic and non-academic institutions. The correlation was deemed significant at p<0.05.

## Results

There were 65 videos analyzed. A total of 22 videos were excluded from the study, while 43 videos that met the inclusion criteria were included. On average, selected videos were available online for 58.65 months. The average video length and number of views were 13.86 and 34953.95, respectively.

The average VPI score for all videos was 22.3 (ranging from 0 to 421). Private users uploaded 33 (76.7%) of the videos. Academic institutions were responsible for publishing 10 (23.3%) of all videos. There was no correlation between LAP-VEGaS scores and view count (P =0.457) or video length (P = 0.702). Videos produced by academic institutions had higher LAP-VEGaS scores than those produced by non-academic institutions. The comparison of different variables among the three video quality-based groups is shown in Table 1.

**Table 1:** Comparison between low-quality, moderate-quality, and high-quality videos based on LAP-VEGaS scores. * Significant p-value LAP-VEGaS: LAParoscopic Surgery Video Educational Guidelines

Variables	Low quality (LAP-VEGaS score 0-6)	Moderate quality (LAP-VEGaS score 7-12)	High quality (LAP-VEGaS score 13-18)	p-value
Mean view count	24344.09	49025.00	28333.33	0.457
Mean length	15.36	11.33	18	0.702
Mean number of likes	27	100.67	165.33	0.000*
Video Power Index	6.8	42.03	17.67	0.21

Table 2 depicts the checklist for evaluating TURP surgical videos’ educational value. The average LAP-VEGaS score was 6.58, and 22 (51.2%), 18 (41.8%), and three (7%) of the videos were classified as having low, medium, and high educational quality, respectively. In general, videos lacked a formal presentation of the case (n=29; 67.4%), graphic aids to emphasize relevant anatomy (n=37; 86%), information on postprocedural outcomes (n=34; 79.1%), and surgical positioning (n=35; 81.4%). The majority of videos had a clear image quality (n=33; 76.7%), demonstrated the surgical procedure partially (n=30; 69.8%), and had English narration (n=22; 51.2%).

**Table 2:** Evaluation of TURP surgical videos using the LAP-VEGaS checklist TURP: transurethral resection of the prostate; LAP-VEGaS: LAParoscopic Surgery Video Educational Guidelines

Variables	Not presented; N (%)	Partially presented; N (%)	Completely presented; N (%)
Authors’ information and video introduction	5 (11.6%)	25 (58.2%)	13 (30.2%)
Formal presentation of the case	29 (67.4%)	8 (18.6%)	6 (14%)
Position of the patient and surgical team	35 (81.4%)	4 (9.3%)	4 (9.3%)
Demonstration of the surgical procedure	6 (14%)	30 (69.8%)	7 (16.2%)
Demonstration of the intraoperative ﬁndings	16 (37.2%)	17 (39.5%)	10 (23.3%)
Outcome of procedure	34 (79.1%)	5 (11.6%)	4 (9.3%)
Associated educational content (diagrams, photos)	37 (86%)	4 (9.3%)	2 (4.7%)
Audio/written commentary in the English language	13 (30.2%)	8 (18.6%)	22 (51.2%)
Image quality is adequate	2 (4.7%)	8 (18.6%)	33 (76.7%)

## Discussion

The utilization of video-based resources presents a highly effective method for acquiring knowledge about surgical techniques, with a special emphasis on minimally invasive endoscopic procedures. According to some studies, YouTube has emerged as the predominant platform for surgical education and training [1, 2, 4].

Despite their extensive use and simple accessibility, most YouTube videos covering surgical procedures lack the scholarly rigor required to be a trustworthy source of medical education. Furthermore, anyone can freely share videos on this platform, which sometimes compromises the video's photography, editing, and recording quality [11]. Without peer evaluation and quality assessment, YouTube is not a reliable educational or informational resource, according to several studies [11, 18, 19]. These findings remind us that when we use surgical videos as an educational tool, we must assess their quality. Also, the findings underscore the imperative for thorough assessment when utilizing surgical videos as educational tools, prompting the development of standards and mechanisms to ensure the educational integrity of digital learning materials.

To date, there is a lack of published studies that have assessed the educational value of TURP surgical videos uploaded on YouTube. Given the potential educational value and enormous influence of these videos, it may be necessary and reasonable for trainees to evaluate their quality.

The LAP-VEGaS score can be used as a video evaluation tool to improve the overall quality of surgical videos on YouTube [15]. Several studies have used the LAP-VEGaS score to assess the usefulness of YouTube videos as a surgical instruction resource. It serves as an exemplary model for the development of educational videos that possess a coherent and systematic framework, specifically designed to facilitate laparoscopic surgical training. Adhering to these guidelines will augment the pedagogical and scholarly merit of surgical videos [16]. Consistent with previous studies, this study showed that there is no correlation between LAP-VEGaS scores and the number of views or the length of videos [2, 8]. According to our study, the educational value of the most popular videos is not the highest. This result implies that a video's popularity alone may not accurately reflect the merit of TURP videos on YouTube. This finding is valuable as it underscores the need for viewers to look beyond mere popularity metrics when assessing the quality and educational value of TURP videos on YouTube. Factors other than popularity, such as content accuracy, relevance, and educational depth, should be considered when determining the merit of educational videos in the medical field.

When reviewing the literature on video sources, the video's source determines its quality; films created by academic institutions tend to have more accurate information and high educational value [14, 20]. This aligns with our findings, which showed that academic institution videos had higher LAP-VEGaS scores than non-academic institution videos. The study showed that academic institutions accounted for a quarter of the uploaded videos, indicating their acknowledgment of YouTube as a platform for spreading knowledge. Private users shared the majority of the videos, demonstrating that surgeons actively contribute by sharing their knowledge and experiences. This video uploader’s distribution is consistent with previous studies about surgical videos on YouTube [21, 22]. These results highlight the collaborative aspect of medical education on YouTube, involving input from both individual experts and institutional channels, and also show that YouTube has emerged as the predominant platform for surgical education and training. 

In this study, the available video content frequently inadequately addressed important surgical knowledge components such as indications, surgical steps, outcomes, and procedure complications. Furthermore, essential aspects such as formal case presentation, description of patient positioning, and educational content like audio or written commentary were missing in the large majority of the evaluated videos. The results obtained were compatible with the literature [23]. These results serve as a reminder for educators to take a cautious approach to suggesting YouTube content as additional learning resources, ensuring they are of high quality before recommending them. It is believed that standardizing the videos uploaded to YouTube can enhance their quality.

Limitations

Our study's primary limitation stems from the limited time frame in which we collected the data. We widely acknowledge that the open nature of the platform subjected data about YouTube videos to dynamic fluctuations. Another limitation is that the research could have included more videos. Since we only included the most popular videos in our study, it is evident that adding more videos will have a disincentive effect. Moreover, we restricted our search criteria to only English-language videos. Consequently, there is a selective bias as authors can publish videos in languages other than English.

## Conclusions

A significant proportion of TURP videos available on the YouTube platform exhibit limited educational value. The order of YouTube search results for a given topic is determined not by quality, but by viewer engagement and popularity. Videos frequently lack comprehensive and in-depth descriptions of surgical operations. The variability in the quality of educational surgical video content is a significant consideration that demands the careful attention of surgical trainees. 

These results demonstrate the need for additional reporting guidelines. To enhance the quality of delivered information and, consequently, the educational experience of students, educational institutions should participate in the sharing of videos on this platform.
